# PANDEMIC CAREGIVING: A LONGITUDINAL ASSESSMENT OF THE TRAJECTORIES AND CORRELATES OF STRESS

**DOI:** 10.1093/geroni/igac059.1259

**Published:** 2022-12-20

**Authors:** Kristin Litzelman, Christina Kim, Margaret Kerr

**Affiliations:** University of Wisconsin-Madison, Madison, Wisconsin, United States; University of Wisconsin-Madison, Madison, Wisconsin, United States; University of Wisconsin-Madison, Madison, Wisconsin, United States

## Abstract

In public health emergencies, caregivers are a crucial but often overlooked human resource. The purpose of this longitudinal study was to assess the well-being of caregivers and non-caregivers over the first 18 months of the COVID-19 pandemic. We used three waves of data from the Survey of the Health of Wisconsin’s COVID-19 Community Impact Survey (Wave 1: May-June 2020; Wave 2: January-February 2021; Wave 3: June 2021; n=2,434 observations of 1,653 unique respondents). Caregivers were identified as those providing care for an adult with an illness or disability. Perceived stress (Global Stress Scale; mean=5.08, SD=4.81) was regressed on caregiver status and covariates in mixed models accounting for repeated measures. On average, caregivers had higher stress than non-caregivers (beta=2.10, p< 0.0001). Across the sample, stress increased between summer 2020 and winter 2021 (mean of 4.8 versus 5.8, p<.01), and lowered somewhat by summer 2021 (mean=5.0, p<.05); this trajectory was similar on average for caregivers and non-caregivers. Respondents who transitioned into a caregiving role during the pandemic had the highest stress (beta=2.55, p< 0.01 compared to non-caregivers). Other factors associated with higher stress (p<.01) include marginalized racial/ethnic identity (beta=1.74), being employed (beta=1.47) or female (beta=0.66), or caregiver having more health conditions (beta=0.22 per condition). Public benefits use and higher self-efficacy were associated with lower stress (betas=-1.18 and -0.30, respectively, p<.01). The findings emphasize the adverse outcomes experienced by caregivers and non-caregivers over the course of the pandemic and highlight potential factors that can inform risk stratification and interventions to support well-being in future crises.

